# Pictorial essay: Coronary artery variants and anomalies

**DOI:** 10.4103/0971-3026.45345

**Published:** 2009-02

**Authors:** Anand M. Rahalkar, Mukund D. Rahalkar

**Affiliations:** Department of Imaging, Sahyadri Speciality Hospital, Pune-411 004, India

**Keywords:** Coronary arteries

## Abstract

CT coronary angiography has helped radiologists understand the variations and anomalies of the anatomy of the coronary arteries and, thus, to alert the cardiologist whenever such an anomaly is present. This can be of immense help to the clinician planning interventional procedures such as stenting, balloon dilatation, or graft surgery, particularly when there are secondary changes of calcification, plaque formation and stenosis.

We present an account and atlas of coronary artery variants and anomalies culled from a database of more than 15000 CT coronary angiographies (CTCA) carried out on a 16-slice Siemens scanner (Somatom 16, Erlangen, Germany) from November 2004 till March 2008 at our institute. Except for a few patients with a prior history of angina, established myocardial infarction, or intervention in the form of coronary stenting or bypass surgery, all the rest underwent the investigation as part of a health check-up package that offered CTCA as an additional investigation.

Over the past few years, several workers have described coronary artery variants and anomalies as identified on catheter angiographies. Angelino *et al*,[1] described various minor anomalies in the branching pattern of coronary arteries and in the location of the cusps and reported an incidence of anomalies in about 1% of the general population. From our own experience with CTCAs, most of which were performed on asymptomatic people rather than on symptomatic subjects, we too are of the opinion that the incidence of coronary variants and anomalies is under 1% in the general population.

After the introduction and establishment of CTCA as an alternative to catheter angiography, a few articles have been published on the detection of such variations and anomalies. Recently, Cademartiri *et al*,[2] have reported 100 (18%) anomalies from 543 consecutive CTCAs done using a 64-slice CT scanner. According to him, catheter angiography cannot detect ectopic openings of coronary arteries since it is only a two-dimensional study. Often, a diagnosis of an anomaly is made when angiography fails to show the normal anatomy. On CTCA, 14 (16.5%) coronary anomalies were detected among the 85 patients who did not have significant coronary artery disease in their study, while 86 coronary anomalies (18.8%) were detected among the 458 patients with significant coronary artery disease. These anomalies included anatomical variations as well as aneurysms.

Variations in coronary anatomy are often seen in association with structural forms of congenital heart disease like Fallot's tetralogy, transposition of the great vessels, Taussig-Bing heart (double-outlet right ventricle), or common arterial trunk.[3] Importantly, coronary artery anomalies are a cause of sudden death in young athletes even in the absence of additional heart abnormalities.[4] Prior knowledge of such variants and anomalies is necessary for planning various interventional procedures.

It is estimated that nearly 5.6% of the total American population could have some kind of coronary anomaly and up to 15% of sudden deaths in young athletes are probably related to these anomalies.[4]

The greatest advantage of CTCA is the high spatial and temporal resolution it provides, so that ectopic origins of anomalous arteries and their paths can be confirmed with greater ease and confidence.

## Variants and anomalies

In the literature there are many terms that have been used to describe variations in coronary anatomy, e.g., abnormal, ectopic, atypical, anomalous, aberrant, accessory, etc.

A) **Variants** refer to simple variations in the structural anatomy.

### Type of dominance

1. The posterior descending artery (PDA) may be supplied by the right coronary artery (RCA); this is referred to as RCA dominance.
2. The PDA may be supplied by the circumflex (CX); this is referred to as left coronary dominance.
3. When both arteries supply the PDA, it is described as co-dominance

### Early branching of coronaries

**Figure 1:**
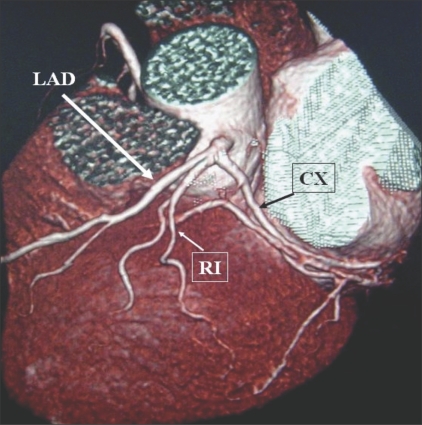
LM trifurcation showing RI: Volume rendered (VRT) image reveals trifurcation of the LM into LAD, CX, and RI. This latter branch is considered by some as either a diagonal or an OM with a high origin

**Figure 2:**
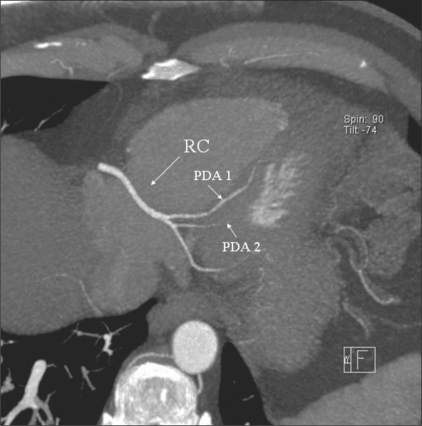
PDA duplication. Axial maximum intensity projection (MIP) image shows duplication of the PDA, with two PDAs (PDA1 and PDA2) arising from the RCA (RC)

**Figure 3:**
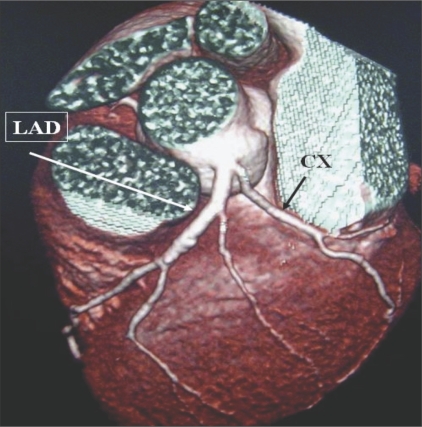
Early division of LM. VRT image shows LM with early division into LAD and CX arteries.

**Figure 4:**
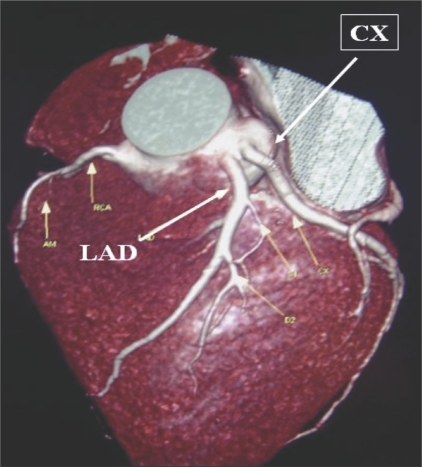
Separate origins: VRT image shows separate origins of the LAD and the CX from the left cusp.

1. The ramus intermedius (RI) is an artery arising between the left anterior descending artery (LAD) and the CX. Some call it a high diagonal (D) or a high obtuse marginal (OM) artery [Figure 1].
2. Early branching of the PDA: when it arises from the RCA before the crux of the heart.
3. Duplication of branches, e.g., two PDAs entering into the septum [Figure 2].
4. Early branching of the left main coronary (LM) into the LAD and the CX [Figure 3] or separate origins of the LAD and the CX from the left aortic cusp [Figure 4].

## Myocardial bridging of LAD

**Figure 5:**
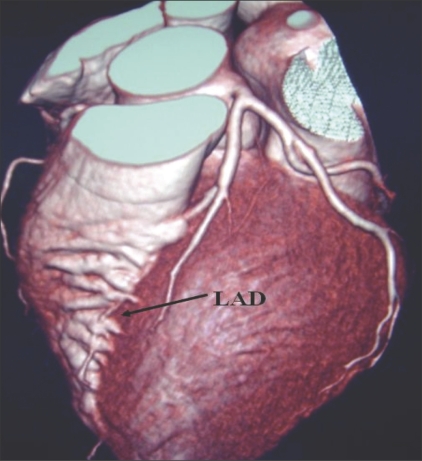
LAD bridging: VRT image shows bridging of the LAD (arrows). The LAD can get submerged in the myocardium of the left ventricle for a variable length (as in this case) when it is termed as myocardial bridging

It can be a normal variant. However, tunnelled LAD is reported in approximately 5% of field deaths among athletes[5] [Figure 5].

B) **Anomalies** are those variations related to the origin and course of the coronary arteries; they may be benign or dangerous (the so-called malignant anomaly). The latter predispose a person to early vascular compromise, ischemia, and fatal infarction. These are far less common (less than 1%).[5]

**Figure 6 (A, B):**
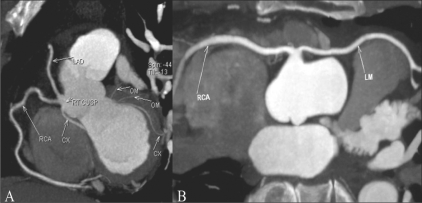
Single coronary artery: MIP image shows a single coronary artery arising from the right cusp (A), with the LM (B) coursing anterior to the right ventricular outflow tract (benign course)

**Figure 7:**
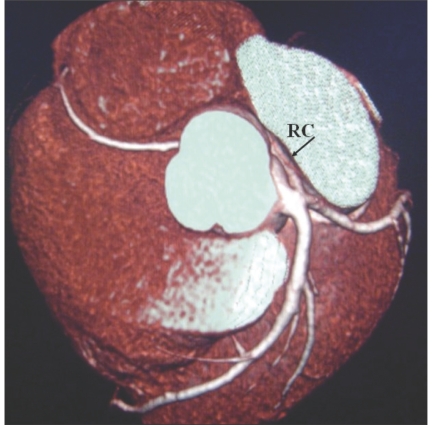
Single coronary artery: All coronaries arise from the left cusp with a single origin and there is a retro-aortic (benign) course of the RCA (RC)

**Figure 8:**
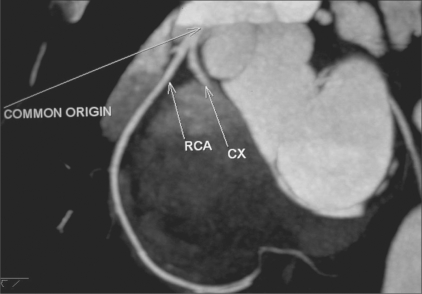
Common origin of the RCA and CX: MIP image shows a common origin of the RCA and CX from the right cusp, the CX coursing posterior to the aorta

**Figure 9:**
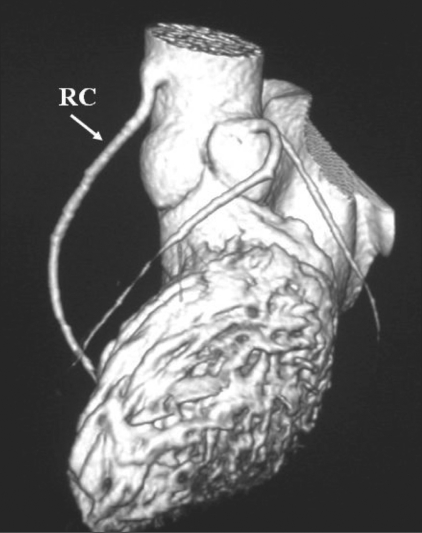
High origin of the RCA: VRT image shows the RCA (RC) arising from the root of the aorta, well above the right cusp

Benign anomalies may be of different types:

Abnormal origin of coronaries:

1. A single coronary arises from the right cusp and divides into the RCA and the LM, with the LM coursing anterior to the RV outflow tract [Figure 6].[6]
2. A single coronary artery arises from the left cusp and divides into the RCA and the LM, with the RCA coursing posterior to the aorta [Figure 7].
3. The CX arises from the right aortic cusp and courses posterior to the aorta [Figure 8].
4. The RCA arises from the aorta superior to the cusp [Figure 9].
5. Miscellaneous (and rarer) anomalies such as congenital hypoplasia or origin of the coronaries from the subclavian or internal mammary arteries.

**Figure 10:**
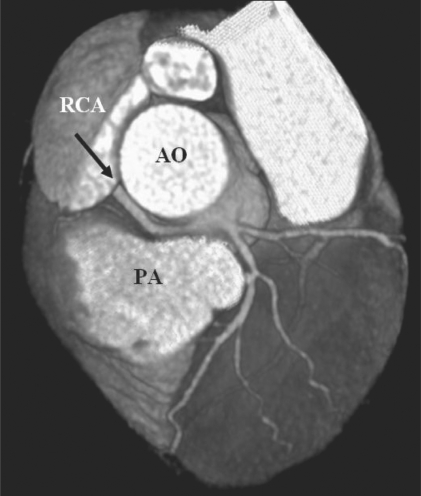
Malignant RCA: VRT image shows a malignant RCA coursing between the aorta and the pulmonary artery

**Figure 11:**
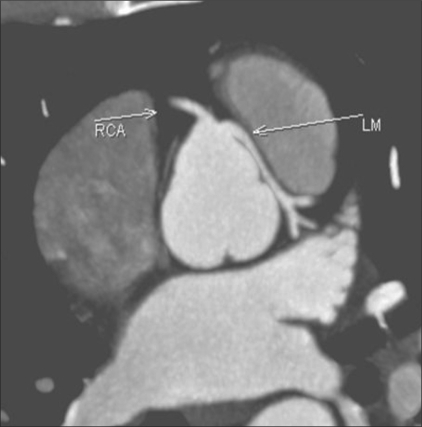
Malignant LM: Axial MIP image reveals a malignant LM taking origin from the right cusp and coursing between the aorta and the pulmonary artery

**Figure 12 (A–D):**
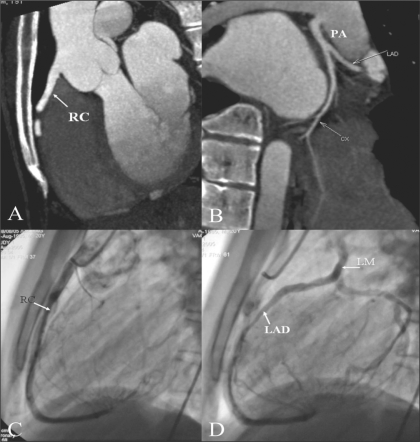
ALCAPA. Sagittal MIP image igure (A) shows normal origin of the RCA (RC) from the right cusp. MIP image (B) reveals the LM originating from the PA — ALCAPA. Lateral coronary angiogram (C) shows a normal RCA (RC). Late phase of the same run (D) shows retrograde filling of the CX, LAD, and then the LM, which eventually opens into the PA

Malignant anomalies include the following:

1. Malignant RCA - This occurs when the RCA arises from the left aortic cusp and courses between the aorta and the PA [Figure 10].
2. Malignant LM - This occurs when the LM arises from the right cusp and courses between the aorta and PA [Figure 11].
  In both situations, the interarterial segment of the artery is subjected to compression during heavy exercise and can cause sudden death in young persons or athletes. Basso[5] considered that an LM originating from the right cusp and crossing between the RCA and the aorta was more dangerous than an RCA taking a similar path, as a larger volume of myocardium is put at risk in the former case. The other mechanisms may be the presence of a slit-like opening or an acute angulation at the origins of such arteries.
3. ALCAPA (anomalous origin of the left coronary artery from the pulmonary artery) [Figures 12], also termed as Bland-White-Garland syndrome (after the names of the persons who first described it in 1933). Usually an isolated anomaly, it is rare and is found in 0.25-0.5% of all congenital heart diseases, causing 90% mortality in the first year of life.[6,7,8] In this condition, the flow in the LM reverses and blood enters the PA due to low pulmonary resistance. This leads to coronary steal and hypoperfusion of the left myocardium, LV dysfunction, and congestive cardiac failure. Another form of this anomaly, with the RCA originating from the PA but shunting blood from the LM is also described.
4. Congenital coronary artery fistula is a rare anomaly. Pelech[9] described a form in which a fistula may occur between any coronary artery and a cardiac chamber or the systemic or pulmonary circulation. As the blood is shunted, the myocardium suffers from ischemia, which may lead to cardiac failure. Most of the fistulae involve the RCA and drain into the RV, RA, or the coronary sinus.
